# Extracellular gentamicin reduces the activity of connexin hemichannels and interferes with purinergic Ca^2+^ signaling in HeLa cells

**DOI:** 10.3389/fncel.2014.00265

**Published:** 2014-09-04

**Authors:** Vania A. Figueroa, Mauricio A. Retamal, Luis A. Cea, José D. Salas, Aníbal A. Vargas, Christian A. Verdugo, Oscar Jara, Agustín D. Martínez, Juan C. Sáez

**Affiliations:** ^1^Departamento de Fisiología, Facultad de Ciencias Biológicas, Pontificia Universidad Católica de ChileSantiago, Chile; ^2^Centro de Fisiología Celular e Integrativa, Facultad de Medicina, Clínica Alemana Universidad del DesarrolloSantiago, Chile; ^3^Instituto Milenio, Centro Interdisciplinario de Neurociencia de Valparaíso, Universidad de ValparaísoValparaíso, Chile

**Keywords:** aminoglycosides, connexins, Cx26, dye uptake, membrane current, intracellular calcium

## Abstract

Gap junction channels (GJCs) and hemichannels (HCs) are composed of protein subunits termed connexins (Cxs) and are permeable to ions and small molecules. In most organs, GJCs communicate the cytoplasm of adjacent cells, while HCs communicate the intra and extracellular compartments. In this way, both channel types coordinate physiological responses of cell communities. Cx mutations explain several genetic diseases, including about 50% of autosomal recessive non-syndromic hearing loss. However, the possible involvement of Cxs in the etiology of acquired hearing loss remains virtually unknown. Factors that induce post-lingual hearing loss are diverse, exposure to gentamicin an aminoglycoside antibiotic, being the most common. Gentamicin has been proposed to block GJCs, but its effect on HCs remains unknown. In this work, the effect of gentamicin on the functional state of HCs was studied and its effect on GJCs was reevaluated in HeLa cells stably transfected with Cxs. We focused on Cx26 because it is the main Cx expressed in the cochlea of mammals where it participates in purinergic signaling pathways. We found that gentamicin applied extracellularly reduces the activity of HCs, while dye transfer across GJCs was not affected. HCs were also blocked by streptomycin, another aminoglycoside antibiotic. Gentamicin also reduced the adenosine triphosphate release and the HC-dependent oscillations of cytosolic free-Ca^2+^ signal. Moreover, gentamicin drastically reduced the Cx26 HC-mediated membrane currents in *Xenopus laevis* oocytes. Therefore, the extracellular gentamicin-induced inhibition of Cx HCs may adversely affect autocrine and paracrine signaling, including the purinergic one, which might partially explain its ototoxic effects.

## INTRODUCTION

Two cells in close contact can exchange metabolites, second messengers and ions through gap junction channels (GJCs; Goldberg et al., 2004). Consequently, GJCs are key elements for diverse coordinated physiological responses of cell communities in most organs. Each GJC is made by the serial docking of two hemichannels (HCs), each one contributed by one of two adjacent cells. In turn, each HC is composed of six protein subunits called connexins (Cxs; Sáez et al., 2003). Undocked HCs are cell membrane channels permeable to ions and small molecules, constituting a communication pathway between the cytoplasm and the extracellular environment (Sáez et al., 2005).

Several mutations in genes coding for Cxs cause congenital prelingual syndromic and non-syndromic hearing loss. About 50% of the hearing loss cases are congenital, being most frequently caused by mutations in the GJB2 gene that encodes Cx26 (Martínez et al., 2009). The remaining 50% of these cases is due to environmental causes, including bacterial and viral infections, acoustic trauma and ototoxic drugs (Resendes et al., 2001). However, it remains unknown whether Cx26 HCs participate in the etiology of acquired hearing loss.

According to the World Health Organization (WHO, 1994), ototoxic drugs are substances of various structures and classes that cause harmful effects in hearing and/or balance organs. This group of molecules includes the aminoglycosides, which are the most common and dangerous ototoxic drugs. Isolated from *Streptomyces* or *Micromonospora*, they are highly hydrophilic antibiotics and have two or more amino groups that confer them a basic nature (Begg and Barclay, 1995). Due to high efficacy and low cost, aminoglycosides are widely used in the treatment of infections caused by aerobic Gram-negative bacteria as well as some mycobacteria (González and Spencer, 1998). However, their use is limited due to negative side effects on kidney and cochlea (for recent reviews, see López-Novoa et al., 2011; Xie et al., 2011). In the inner ear, the ototoxicity induced by aminoglycosides culminates in the destruction of the cochlear sensory hair cells (Huth et al., 2011).

The most extensively studied aminoglycoside is gentamicin. Among the proposed mechanisms for its ototoxicity and nephrotoxicity, it has been suggested the generation of reactive oxygen species and nitric oxide (Sha and Schacht, 1998; Basnakian et al., 2002; Hong et al., 2006; Choung et al., 2009). The routes of gentamicin uptake in the inner ear structures are not fully understood, but several mechanisms have been proposed, including mechanotransducer channels (METs) located on stereocilia of hair cells, endocytosis in the apical or basolateral membranes of the organ of Corti, TRP channels or adenosine triphosphate (ATP) receptors (Huth et al., 2011). Moreover, it has been reported that MET channel opening is required to induce gentamicin toxicity in hair cells, suggesting an intracellular toxicity mechanism (Alharazneh et al., 2011). In addition, early studies suggested that cochleotoxicity of gentamicin is an excitotoxic process involving the activation of NMDA receptors (Basile et al., 1996), which might also be explained by the generation of free radicals (Sha and Schacht, 1998). In contrast, aminoglycosides have been shown to block a variety of ionic channels, such as hair cell METs (Jaramillo and Hudspeth, 1993), acetylcholine receptors (Blanchet et al., 2000) and purinergic ionotropic channels (Bongartz et al., 2010), all of which might partially explain their toxic effects. Purinergic signaling is one of the main mechanisms of paracrine signaling in the cochlea and has been associated to activation of K^+^ recycling in cochlear supporting cells, being fundamental for the normal functioning of this sensory organ (Zhu and Zhao, 2010).

More than a decade ago, Todt et al. (1999) demonstrated that extracellular gentamicin inhibits gap junctional electrical coupling through free radical production in isolated cochlear supporting cells. However, the possible effects of gentamicin on HCs remain unknown, and the mechanisms responsible for its effects on GJCs are still not fully understood. In this work, we show that gentamicin applied to the extracellular media reduces the functional activity of HCs in a Cx composition-independent way. However, the same gentamicin concentration has no effect on intercellular dye coupling through GJCs. Gentamicin also reduces the oscillations of Cx HC-dependent cytosolic Ca^2+^ signals elicited by extracellular ATP in HeLa-Cx26 cells. Moreover, we found that gentamicin, like other GJC/HC blockers, reduces ATP release in HeLa-Cx26 cells, triggered by divalent cation-free solution (DCFS) or by UTP, a purinergic receptor agonist. Since Cx HCs play a relevant role as cellular membrane pathways for autocrine/paracrine signaling including the purinergic signaling of the inner ear (Zhao et al., 2005), gentamicin-induced Cx HC dysfunction may adversely affect these pathways, partially explaining the ototoxicity induced by this antibiotic through an extracellular mechanism.

## MATERIALS AND METHODS

### REAGENTS

Ethidium (Etd) bromide, LaCl_3_, adenosine triphosphate disodium (Na_2_ATP), cyclopiazonic acid (CPA, sarcoplasmic reticulum Ca^2+^-pump inhibitor), carbonyl cyanide 3-chlorophenylhydrazone (CCCP, H^+^ ionophore and uncoupler of oxidative phosphorylation in mitochondria), U73122 [Phospholipase C (PLC) inhibitor], carbenoxolone (CBX) and streptomycin were obtained from Sigma-Aldrich (St. Louis, MO, USA). Fura-2-AM was obtained from Molecular Probes (Eugene, OR, USA) and gentamicin sulfate from Invitrogen Life Technologies (Carlsbad, CA, USA). Polyclonal anti-P2Y_2_, -P2Y_4_, and -P2Y_6_ receptor antibodies, as well as goat anti-rabbit and anti-mouse secondary antibodies conjugated to horseradish peroxidase, were obtained from Santa Cruz Biotechnology Inc. (Santa Cruz, CA, USA). The mimetic peptide Gap26 (sequence: N-VCYDKSFPISHVR-C) was synthesized by Beijing SBS Genetech Co. Ltd. (Beijing, China).

### GFP-TAGGED Cx26 CONSTRUCT IN pcDNA3.1

The rCx26-GFP fusion protein (rCx26-GFP) was generated as described previously by Jara et al. (2012). The coding region of rat Cx26 (rCx26, NM_001004099.1) was subcloned into pcDNA3.1/CT-GFP-TOPO (Invitrogen Life Technologies, Carlsbad, CA, USA), according to the manufacturer’s instructions. The coding region of the construct was fully sequenced.

### CELL CULTURE

HeLa-Parental cells were obtained from ATCC (CCL-2; ATCC, Rockville, MD, USA) and were stably transfected with the rCx26-GFP construct using Lipofectamine 2000 (Invitrogen Life Technologies, Carlsbad, CA, USA), according to the manufacturer’s instructions. HeLa cells stably expressing rCx26 (NM_001004099.1) were kindly provided by Dr. Bruce Nicholson (Department of Biochemistry at the University of San Antonio, San Antonio, TX, USA), while HeLa cells stably expressing mouse Cx26 (mCx26, NM_008125.3), Cx43 (mCx43, NM_010288.3), or Cx45 (mCx45, NM_001159383.1) were kindly provided by Dr. Klaus Willecke from the LIMES Institute (Bonn Universität, Germany). All cell lines were grown at 37°C and 5% CO_2_ in DMEM supplemented with 10% fetal bovine serum (GIBCO, Invitrogen), 100 U/ml penicillin, 100 μg/ml streptomycin sulfate, and 0.5 μg/ml puromycin to select transfected cells. HeLa rCx26-GFP cells were selected with 500 μg/ml G418. All cell lines were used 48 h after seeding, and all antibiotics used for selection and maintenance were not included during this period of time. Untransfected HeLa-Parental cells were used as control.

### HC ACTIVITY

The HC activity was evaluated using the Etd uptake method as described (Figueroa et al., 2013). In brief, sub-confluent HeLa cells grown on glass coverslips were washed twice with recording solution [in mM: NaCl (148); KCl (5); CaCl_2_ (1.8); MgCl_2_ (1); glucose (5); HEPES (5), pH = 7.4] containing 5 μM Etd. Basal fluorescence intensity from the nucleus of each cell was recorded for 5 min, using an Olympus BX 51W1I upright microscope (Olympus America Inc., Center Valley, PA, USA). Next, cells were washed three times with (Ca^2+^/Mg^2+^-free) DCFS and the fluorescence intensities of the nuclei were recorded. At the end of each experiment, the Cx HC blocker La^3+^ was added to confirm HC mediated Etd uptake (Contreras et al., 2002; Retamal et al., 2007). Dye uptake of each cell was digitally photographed using a CCD monochrome camera (CFW-1310M; Scion; Frederick, MD, USA). Images were captured every 30 s (exposure time = 30 ms, gain = 0.5). Metafluor software (version 6.2R5, Universal Imaging Co., Downingtown, PA, USA) was used for data acquisition and off-line image analysis. The fluorescence intensity of at least 30 cells per experiment was averaged and plotted against time (expressed in minutes). Lastly, the slope, here called Etd uptake rate, was calculated using Microsoft Excel software and expressed as arbitrary units per minutes (AU/min).

### hCx26 cRNA PREPARATION AND INJECTION INTO *Xenopus laevis* OOCYTES

The plasmid pOocyte-Cx26, containing human Cx26 cDNA (hCx26), was kindly provided by Dr. Guillermo Altenberg (Texas Tech University Health Sciences Center, Lubbock, TX, USA). cRNA coding for hCx26 was prepared as previously described (Figueroa et al., 2013). To reduce expression of endogenous Cx38, an antisense oligonucleotide directed against Cx38 was used. After the injection of the cRNA, Oocytes were maintained in Barth’s solution [in mM: NaCl (88); KCl (1); CaCl_2_ (5); MgCl_2_ (0.8); HEPES (10), pH = 7.4] supplemented with 0.1 mg/ml gentamicin and 20 units/ml of penicillin-streptomycin.

### MEMBRANE CURRENT VIA HCs

Dual whole cell voltage clamp recordings of *X. laevis* oocytes injected with hCx26 cRNA were carried out as described (Retamal et al., 2011) using a two electrode voltage clamp amplifier for oocytes (Warner Instruments, model OC-725C) connected to a digital-to analog converter (Molecular Devices, model DigiData 1440A). ND96 medium [in mM: NaCl (96); KCl (2); CaCl_2_ (1.8); MgCl_2_ (1); HEPES (10), pH = 7.4] was used as bath solution in all experiments. Recording pipettes were filled with 3 M KCl. For data acquisition and analysis, the pClamp 10 software was used. Currents were measured after 15 s rectangular voltage pulses, ranging from -60 to +40 mV, in 10 mV steps with a holding potential of -60 mV and 10 s intervals between pulses. Female *X. laevis* were obtained from the animal facility of Universidad de Chile, and the Commission of Bioethics and Biosafety of the Universidad del Desarrollo approved the experimental protocols.

### INTRACELLULAR Ca^2+^ SIGNAL

The intracellular Ca^2+^ signal was evaluated as described (Figueroa et al., 2013). The intracellular Ca^2+^ signal was monitored in Fura-2-AM (5 μM) loaded HeLa cells grown on glass coverslips, and recording solution described above for dye uptake experiments was used. Fluorescence from regions of interest (ROI’s) covering single Fura-2 loaded cells was determined at excitation wavelengths of 340 and 380 nm, while fluorescence emission was collected at 510 nm every 3 s using an Olympus BX 51W1I upright microscope. The intracellular Ca^2+^ signal was calculated as R = F_340__nm_/F_308__nm_, and the background was subtracted. Ca^2+^ transients were evoked by extracellular application of ATP and signals obtained were averaged, including at least 30 cells per experiment. Subsequently, the area under curve (AUC) and duration (measured as the time between the first increase in Ca^2+^ signal until the return to baseline) were calculated and represented graphically.

### EXTRACELLULAR ATP MEASUREMENT

Adenosine triphosphate release from HeLa-rCx26 cells was determined using the ATP bioluminescence assay kit (Sigma) in combination with a spectrofluorometer (Jasco Corp., FP-63000, Tokyo, Japan). HeLa-rCx26 cells were seeded into 60 mm culture plates 24 h before each experiment or until they reached 70% confluence. For extracellular ATP measurements, the culture medium was removed and cells were washed twice with DCFS or Ca^2+^/Mg^2+^-containing solution. Then, cells were incubated for 5 min in 500 μl of DCFS or treated with 100 μM UTP in Ca^2+^/Mg^2+^-containing solution to induce ATP release. Subsequently, the 500 μl of extracellular solution were carefully collected to avoid damaging the cells, and ATP content was determined immediately using the luciferin/luciferase bioluminescence assay. To this end, 50 μl of the extracellular solution were mixed in the cuvette with 50 μl of luciferin/luciferase reagent, and the average light signal was measured for 10 s with a spectrofluorometer (Jasco Corp., FP-63000, Tokyo, Japan). The different reagents used in these experiments were diluted in 500 μl of DCFS or Ca^2+^/Mg^2+^ solution resulting in the following concentrations: 200 μM gentamicin and 100 μM CBX. The ATP concentration was determined by using a luminescence standard curve, and data were normalized by the total cellular protein present in the plate. When CBX and gentamicin were used, they were included from the first wash at the indicated concentration.

### WESTERN BLOT ANALYSES

Relative levels of proteins were assayed by Western blot analyses as described (Figueroa et al., 2013). Blots were incubated with primary polyclonal anti-P2Y_2_, -P2Y_4_, or -P2Y_6_ antibodies overnight at 4°C, followed by five washes with TBS, 1% Tween-20 buffer. Then, they were incubated with goat anti-rabbit secondary antibodies and conjugated with horseradish peroxidase. The immunoreactivity was detected by electrogenerated chemiluminescence (ECL) using the SuperSignal kit (Pierce, Rockford, IL, USA) according to the manufacturer’s instructions. Blots were also developed with α-tubulin, used as loading control.

### DYE COUPLING

The functional state of GJCs was evaluated as described (Figueroa et al., 2013). Briefly, confluent HeLa cell cultures grown on coverslips were used in each experiment. Single cells were iontophoretically microinjected with a glass micropipette filled with 75 mM Etd in water or Lucifer yellow (LY, 5% w/v in 150 mM LiCl). Dye coupling index was calculated as the mean number of cells to which the dye spread occurred. All microinjections were performed in ${HCO}_{3}^{-}$-free F-12 medium buffered with 10 mM HEPES (pH 7.4) containing 200 μM La^3+^ to avoid cell leakage of the microinjected dye via HCs. Fluorescent cells were observed using a Nikon inverted microscope equipped with epifluorescence illumination (Xenon arc lamp) and Nikon B filter to LY (excitation wavelength 450–490 nm; emission wavelength above 520 nm) and XF34 filter to Etd fluorescence (Omega Optical, Inc., Brattleboro, VT, USA). Photomicrographs were obtained using a CCD monochrome camera (CFW-1310M; Scion; Frederick, MD, USA). In all experiments, dye coupling was tested by injecting a minimum of 14 cells.

### STATISTICS

Statistical analysis was performed using GraphPad Prism 5 software for Windows (GraphPad Software, San Diego, CA, USA). Data sets (means ± SEM) were compared using one-way analysis of variance (ANOVA) followed by a Tukey’s post-test or Student’s *t*-test for pair-wise comparisons.

## RESULTS

### GENTAMICIN INHIBITS HC ACTIVITY ELICITED BY EXTRACELLULAR DIVALENT CATION-FREE SOLUTION

**FIGURE 1:**
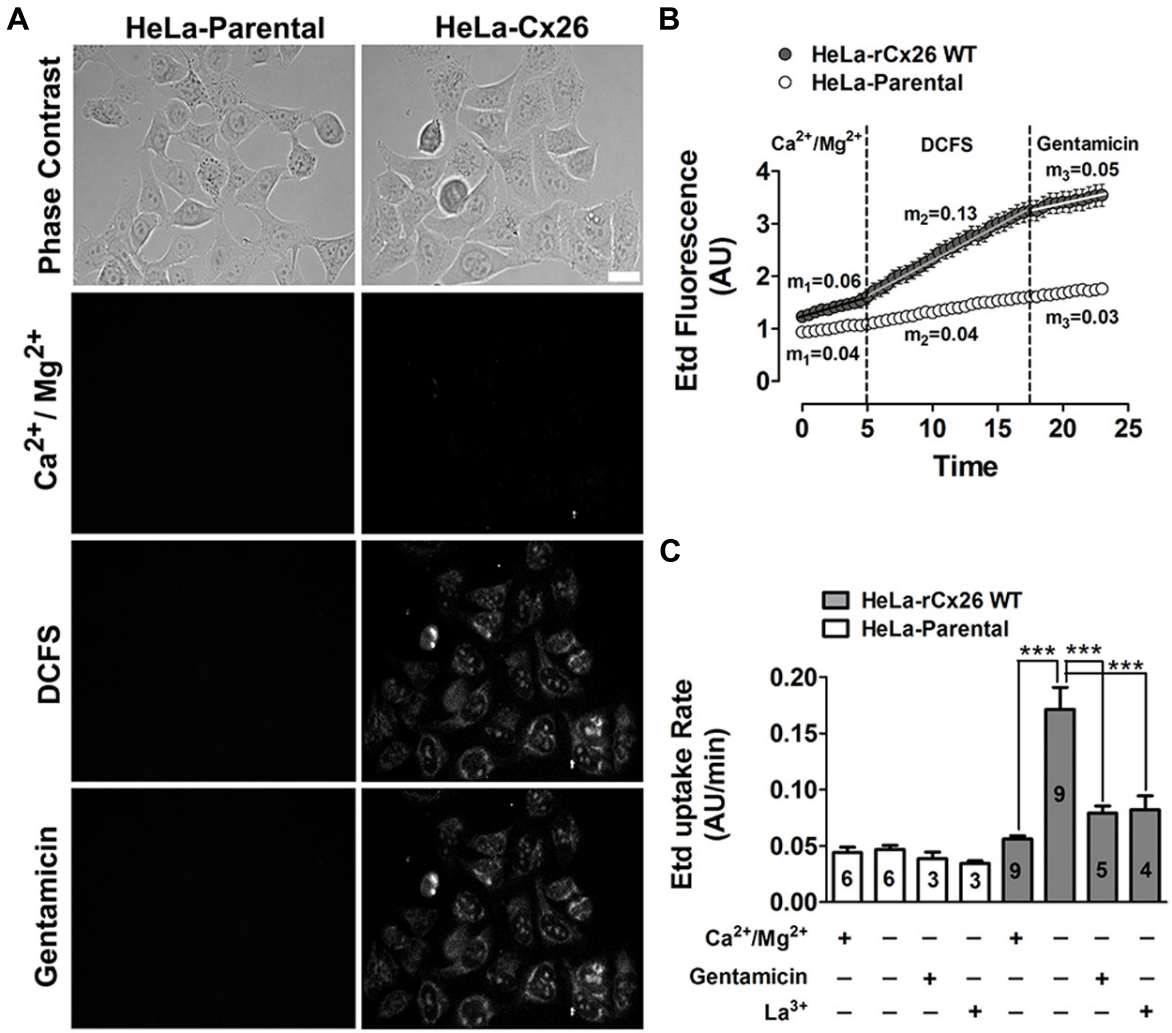
**Gentamicin blocks the ethidium uptake induced by a divalent cation-free solution (DCFS) in HeLa-Cx26 cells.** The ethidium (Etd) uptake was evaluated in HeLa-Parental or -Cx26 cells bathed with divalent cation (Ca^2+^/Mg^2+^) or DCFS solution in time-lapse measurement experiments. **(A)** Representative fluorescent fields showing Etd florescence of HeLa-Parental or -rCx26 cells incubated in saline solution containing 5 μM Etd, under control conditions (Ca^2+^/Mg^2+^, 5 min), after exposure to DCFS (10 min) or after 5 min exposure to DCFS containing 200 μM gentamicin. Scale bar, 40 μm. **(B)** Representative time-lapse experiments showing Etd uptake in HeLa-Cx26 and -Parental cells under control conditions (Ca^2+^/Mg^2+^, first 5 min), after exposure to DCFS solution followed by the application of 200 μM gentamicin (last 5 min). *m*_1_, *m*_2_, *m*_3_ = average slope. Measurements were taken every 30 s as fluorescence emission intensity of Etd bound to DNA and referred as fluorescence intensity expressed in arbitrary units (AUs). Each value corresponds to the mean ± SEM of at least 30 cells. **(C)** Etd uptake rate of Hela-Parental or -rCx26 cells measured under control conditions and after exposure to DCFS with or without 200 μM La^3+^ or 200 μM gentamicin. Data are presented as means ± SEM, the digit within each bar corresponds to the number of independent experiments under that condition. ***P < 0.001.

The functional state of HCs was determined by measuring the cellular uptake of Etd. At a concentration of 5 μM, Etd diffuses across the cell membrane preferentially via HCs (Schalper et al., 2008). Once in the intracellular space, it binds to nucleic acids and emits fluorescence that is proportional to the HCs activity (Contreras et al., 2002; Schalper et al., 2008; Orellana et al., 2010). Etd uptake was measured during 5 min in HeLa-Parental and HeLa-rCx26 cells exposed to a saline solution with physiologic extracellular divalent cation concentrations (**Figure 1A**, Ca^2+^/Mg^2+^). In HeLa-rCx26 cells, the fluorescence intensity was slightly higher than in HeLa-Parental cells (**Figure 1B**, Ca^2+^/Mg^2+^), which do not express Cx26 HCs. In order to increase the open probability of rCx26 HCs, cells were bathed in DCFS (Jara et al., 2012). A rapid increase in fluorescence intensity was measured in HeLa-rCx26 but not in HeLa-Parental cells (**Figures 1A,B**, DCFS). Lastly, the application of 200 μM gentamicin to the bath solution reduced the DCFS-induced Etd uptake in HeLa-rCx26 cells to values similar to those recorded in the presence of physiologic extracellular Ca^2+^/Mg^2+^ concentrations either in HeLa-rCx26 or -Parental cells (**Figure 1B**, gentamicin).

**FIGURE 2:**
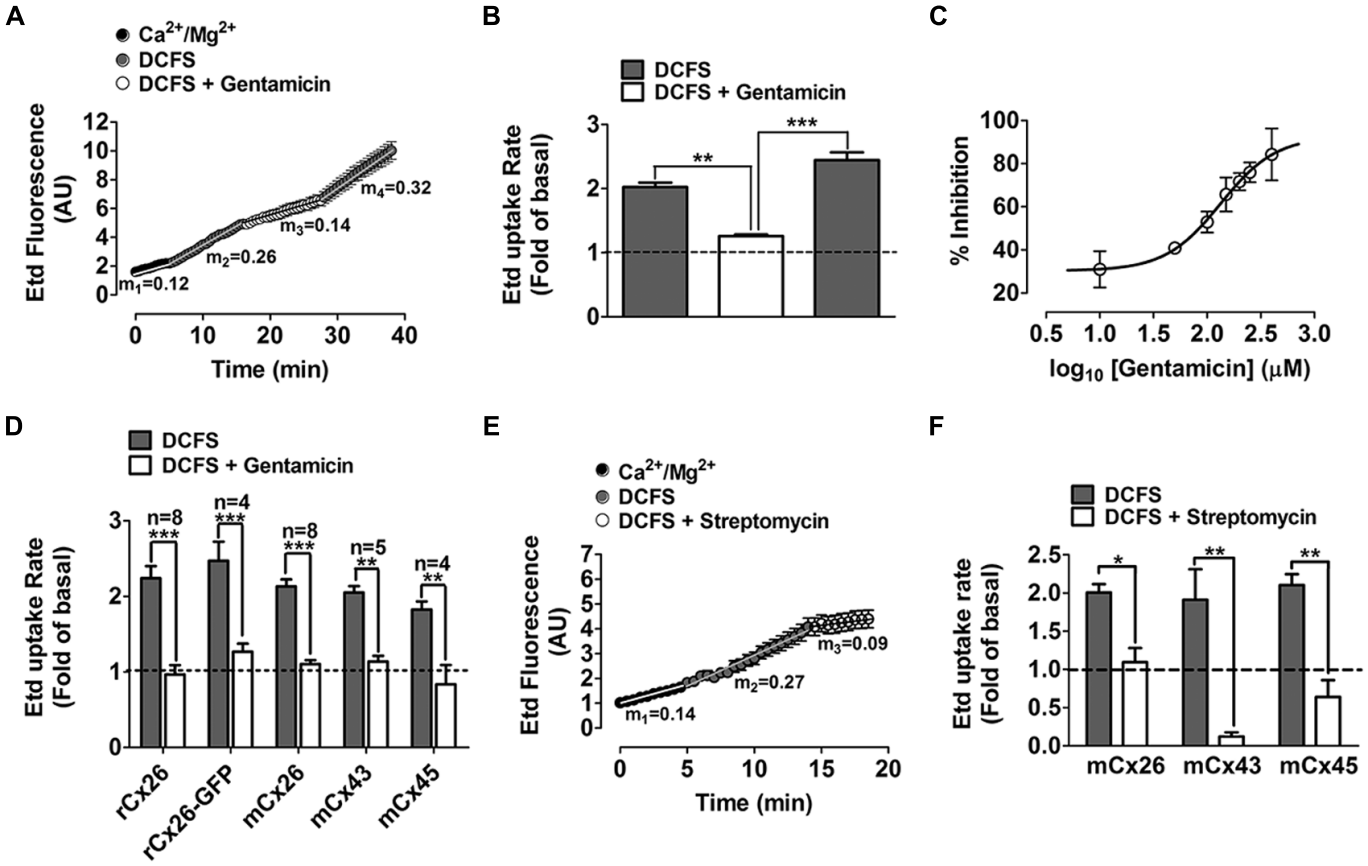
**Gentamicin blocks connexin26 hemichannels in a reversible- and concentration-dependent manner and also blocks connexin43 and connexin45 hemichanels. (A)** Representative time-lapse experiment showing ethidium (Etd) uptake in cells transfected with rat connexin26 (HeLa-rCx26 cells) under control conditions (Ca^2+^/Mg^2+^), after exposure to DCFS, followed by 5 min exposure to DCFS containing 200 μM gentamicin and washout of gentamicin with DCFS (last 10 min), *m*_1_, *m*_2_, *m*_3_, *m*_4_ = average slope. **(B)** Average Etd uptake rate (experiments as shown in **A**); data were normalized to the control Etd uptake level (dotted line). **(C)** Concentration-response curve showing the inhibition of DCFS-induced Etd uptake prompted by different gentamicin concentrations in HeLa-rCx26 cells. Each point represents the mean ± SEM (*n* = 3). Values were normalized to the maximal response in the absence of gentamicin and were included in the Hill equation, IC_50_ = 133.4 ± 1.1 μM, *R*^2^ = 0.95. **(D)** Etd uptake rates of HeLa cells expressing rat Cx26 (rCx26), mouse Cx26 (mCx26), rat Cx26-GFP (rCx26-GFP), mouse Cx43 (mCx43) or mouse Cx45 (mCx45), after exposure to DCFS followed by 5 min exposure to DCFS containing 200 μM gentamicin. Data were normalized to the Etd uptake value measured under control conditions (Ca^2+^/Mg^2+^ solution, dotted line) and are presented as mean ± SEM (*n* = 3). **(E)** Representative time-lapse curve showing Etd uptake in HeLa-rCx26 cells in control conditions and after exposure to DCFS followed by 200 μM streptomycin, *m*_1_, *m*_2_, *m*_3_ = average slope. **(F)** DCFS-induced Etd uptake normalized to control uptake in absence (gray bar) or presence (white bar) of 200 μM streptomycin of HeLa cells expressing rCx26, mCx43, or mCx45. Values recorded in at least 30 cells per experiment were included (*n* = 3). *P < 0.05, **P < 0.01, ***P < 0.001.

The basal Etd uptake rate of HeLa-rCx26 cells bathed with saline solution containing divalent cations was slightly higher than that of HeLa-Parental cells (*m*_1_ = 0.06 AU/min for HeLa-rCx26 v/s *m*_1_ = 0.04 AU/min for HeLa-Parental; **Figure 1B** Ca^2+^/Mg^2+^ first 5 min and C), which might result from basal activity of rCx26 HCs. However, the Etd uptake and Etd uptake rate of HeLa-rCx26 cells was ~two fold higher when bathed with DCFS than in the presence of extracellular divalent cations (**Figures 1B,C**; *m*_1_ = 0.06 AU/min v/s *m*_2_ = 0.13 AU/min), whereas the Etd uptake rate of HeLa-Parental cells in DCFS remained indistinguishable from the rate measured in the presence of physiological concentrations of divalent cations (**Figures 1B,C**, Ca^2+^/Mg^2+^ v/s DCFS). Then, the Etd uptake was drastically inhibited by 200 μM gentamicin (**Figure 1B**) and the Etd uptake rate values changed from *m*_2_ = 0.13 AU/min to *m*_3_ = 0.05 AU/min in Hela-rCx26 cells, but was not affected in HeLa-Parental cells (**Figure 1C**, DCFS + gentamicin). Similar results were obtained in HeLa-rCx26 cells bathed with DCFS and treated with 200 μM La^3+^ (**Figure 1C**), a widely used Cx HC blocker (Sáez et al., 2003). The gentamicin-induced Etd uptake rate inhibition in Hela-rCx26 cells was found to be reversible because it was restored by replacing the bath solution with gentamicin-free DCFS (**Figures 2A,B**). The gentamicin-induced inhibition of rCx26 HCs occurred in a concentration-dependent manner, being 133.4 ± 1.1 μM the concentration that induced 50% Etd uptake rate inhibition (IC50; **Figure 2C**). Gentamicin also inhibited Etd uptake in HeLa-rCx26GFP cells as well as in mCx26, mCx43, or mCx45 bathed in DCFS (**Figure 2D**). In addition, streptomycin (Strep 200 μM), another aminoglycoside antibiotic, also inhibited the Etd uptake of HeLa-rCx26 (**Figures 2E,F**), -mCx43 and -mCx45 cells in DCFS (**Figure 2F**). In HeLa-mCx45 cells, Etd uptake was reduced to values below the ones recorded under control condition (**Figure 2F**), suggesting that these HCs present a higher open probability under basal condition.

### GENTAMICIN INHIBITS THE CELL MEMBRANE CURRENT MEDIATED BY Cx26 HCs

**FIGURE 3:**
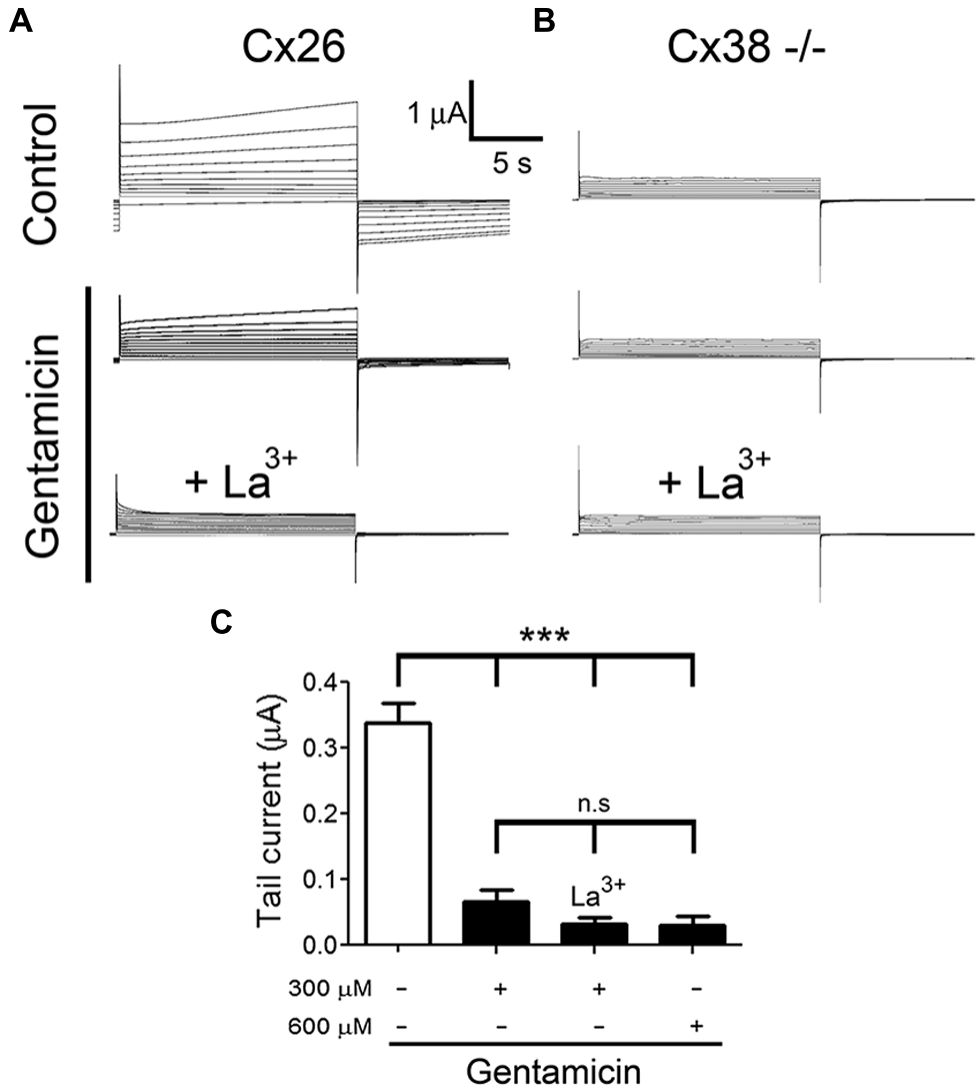
**Gentamicin inhibits the membrane current mediated by human connexin hemichannels.** The membrane current of *Xenopus laevis* oocytes injected with connexin38 antisense oligonucleotide and human connexin (Cx26) cRNA was evaluated in whole cell modality using two electrodes. **(A)** Currents induced by depolarization from -60 to +40 mV (10 mV steps, for 15 s) under control condition (upper panel) or in the presence of 300 or 600 μM gentamicin (middle panel) and 300 μM gentamicin plus 200 μM La^3+^ (lower panel). **(B)** Currents induced by depolarization from -60 to +40 mV in oocytes non-injected with Cx26 cRNA under control conditions (upper panel) or in the presence of 300 or 600 μM gentamicin (middle panel) and 300 μM gentamicin plus 200 μM La^3+^ (lower panel). **(C)** Average maximal tail currents from 5 oocytes in the presence or absence of 300 or 600 μM gentamicin and 300 μM gentamicin plus 200 μM La^3+^. ***P < 0.005 and n.s: not significant.

**FIGURE 4:**
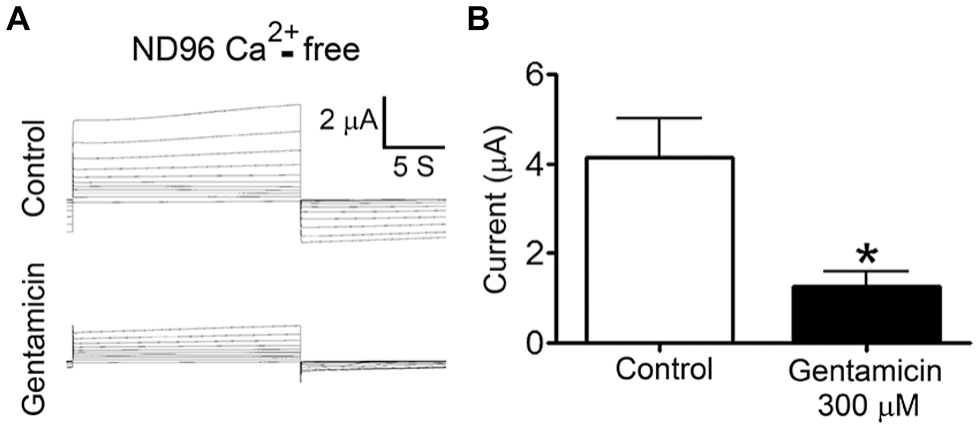
**Gentamicin inhibits the membrane current mediated by human connexin26 hemichannels in nominal Ca^2+^-free solution.** The membrane current of *Xenopus laevis* oocytes injected with connexin38 antisense oligonucleotide and human connexin26 cRNA was evaluated in whole cell modality using two electrodes in ND96 without Ca^2+^ and Mg^2+^. **(A)** Currents induced by depolarization from -60 to +40 mV (10 mV steps, for 15 s) under control conditions (upper panel) or in the presence of 300 μM gentamicin (lower panel). **(B)** Average maximal currents at +40 mV from 5 oocytes in the presence or absence of 300 μM gentamicin. *P < 0.01.

To test whether gentamicin inhibits Cx26 HCs, we recorded membrane currents generated by the application of rectangular command voltages under whole cell dual voltage clamp in *X. laevis* oocytes expressing hCx26. Endogenous oocyte Cx38 expression was inhibited by using specific antisense. Forty eight hours after the cRNA injection, oocytes were depolarized from -60 to +40 mV (10 mV steps) for 15 s (**Figure 3A**). Under control conditions (ND96 solution containing 1.8 mM Ca^2+^), the activation of an outward current was evident (at voltages above 0 mV) followed by a tail current upon repolarization to -60 mV (**Figure 3A**). These currents were virtually absent in oocytes injected with Cx38 antisense oligonucleotide (**Figure 3B**), indicating this was mediated by activated hCx26 HCs. Then, cells were treated for 3–5 min with 300 μM gentamicin applied in the bath, and both the maximal and tail currents were drastically reduced (**Figure 3A**, middle) and completely abolished by the subsequent addition of La^3+^ (**Figure 3A**, bottom). To minimize contamination with endogenous currents, we measured the maximal current at -60 mV before the depolarization at +40 mV. It was observed that gentamicin-induced a concentration-dependent decrease of maximal tail current (**Figure 3C**). Under control conditions, the maximal tail current recorded was 0.34 ± 0.03 μA, and after the addition of 300 or 600 μM gentamicin it was 0.07 ± 0.02 and 0.03 ± 0.02 μA, respectively (**Figure 3C**). Similar results were obtained in presence of 200 μM La^3+^ in the extracellular solution (**Figure 3C**). In the absence of extracellular Ca^2+^, outward currents generated with the different command voltages were more prominent than those recorded in bath solution containing 1.8 mM Ca^2+^ (**Figure 4A**). In the absence of extracellular Ca^2+^, 300 μM gentamicin inhibited the membrane currents (**Figure 4A**) by ~70% (from 4.15 ± 0.87 to 1.28 ± 0.32 μA; **Figure 4B**).

### GENTAMICIN REDUCES THE ATP-INDUCED Ca^2+^ SIGNAL IN HeLa-rCx26 CELLS

Since HCs have been suggested to play a major role in inner ear purinergic signaling (Zhao et al., 2005) and because inhibition of Cx43 HCs reduces the intracellular Ca^2+^ signals elicited by bradykinin (De Bock et al., 2012), we tested whether gentamicin affects the ATP-induced Ca^2+^ signals in HeLa-rCx26 cells.

**FIGURE 5:**
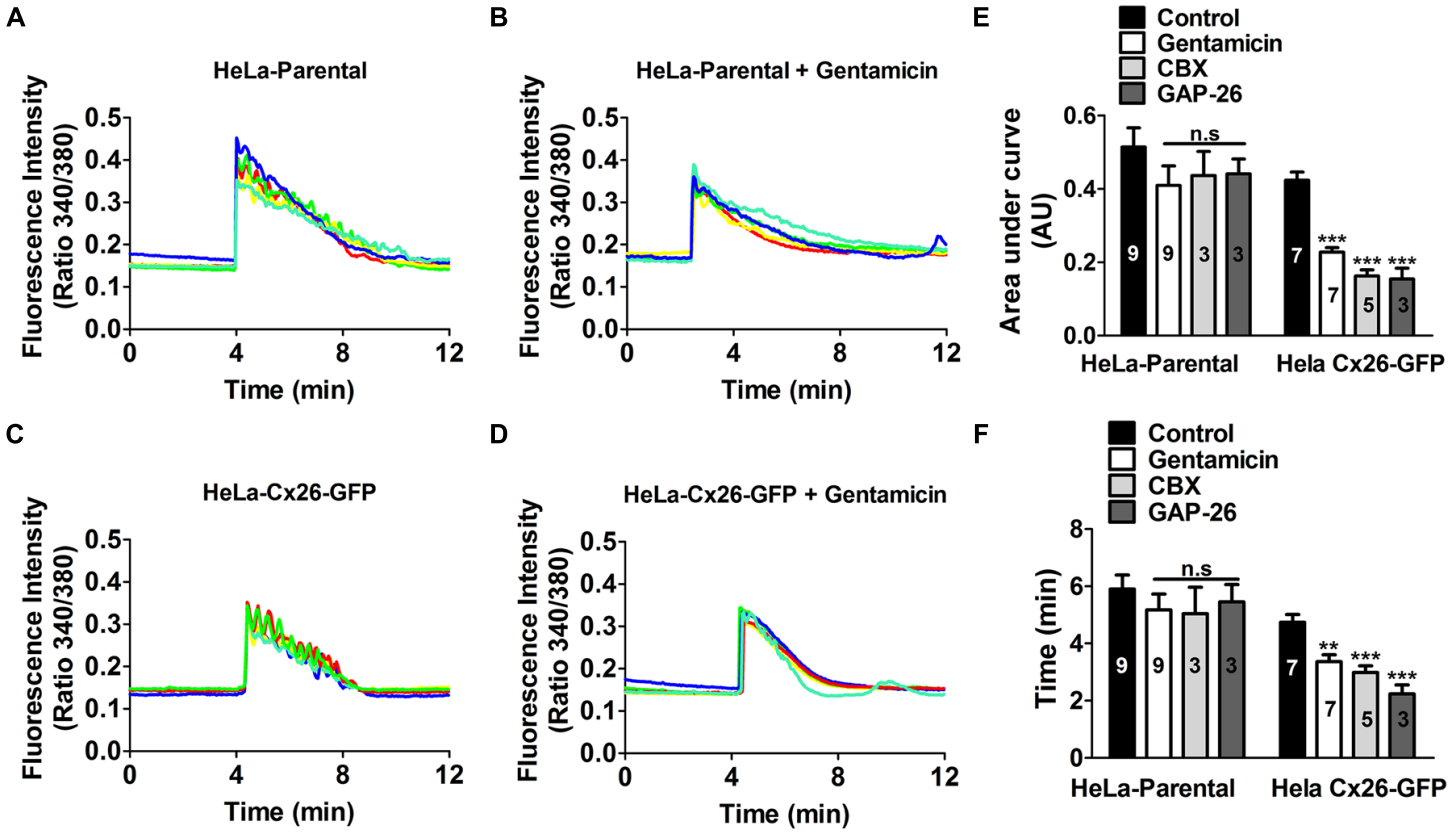
**Gentamicin and hemichannel blockers reduce the ATP-evoked Ca^2+^ signal in HeLa-rCx26GFP cells.** HeLa-Parental cells or HeLa cells stably transfected with rCx26-GFP were loaded with Fura-2 and then stimulated with bath application of 10 μM ATP. **(A)** Time course of ATP-evoked intracellular Ca^2+^ signal under control conditions in HeLa-Parental cells. **(B)** Time course of ATP-evoked intracellular Ca^2+^ signal in HeLa-Parental cells pre-incubated for 5 min with 200 μM gentamicin. Five representative cells are shown in each case. **(C)** Time course of ATP-evoked intracellular Ca^2+^ signal under control conditions in HeLa-rC26-GFP cells. **(D)** Time course of ATP-evoked intracellular Ca^2+^ signal in HeLa-rC26-GFP cells pre-incubated for 5 min with 200 μM gentamicin. **(E)** Compiled data showing the amplitude of Ca^2+^ signal changes evoked by extracellular ATP (mean ± SEM) in HeLa parental and HeLa-rCx26GFP, measured as area under curve expressed as AU in control conditions or after 5 min preincubation with 200 μM gentamicin, 10 min with 100 μM carbenoxolone (CBX) or 20 min with 200 μM GAP-26. **(F)** Duration (time), from the first Ca^2+^ signal increase until the return to baseline (mean ± SEM) in HeLa-Parental and HeLa-rCx26GFP in control conditions or after 5 min preincubation with 200 μM gentamicin, 10 min with 100 μM CBX or 20 min with 200 μM GAP-26. For calculations of the average traces (as shown in A) 40 cells per experiment were included. The number of independent experiments is indicated in each bar. **P < 0.01, ***P < 0.001.

Gentamicin was applied to the bath solution 5 min prior to stimulation with extracellular ATP in presence of 1.8 mM [Ca^2+^]_0_. Fura-2 loaded HeLa-rCx26-GFP cells were used to facilitate identification of cells expressing Cx26 and 10 μM ATP was applied in all experiments. **Figure 5** shows a representative experiment including five representative cells in each record. In all cells, the application of 10 μM ATP induced a fast initial increase in Ca^2+^ signal. In HeLa-Parental cells, small oscillations with ~5 s intervals were superimposed with a sustained Ca^2+^ signal increase that decayed progressively over time (**Figure 5A**). In contrast, in Hela-rCx26-GFP cells the oscillations were more evident than in parental cells (**Figure 5C**). The ATP induced-Ca^2+^ signal increases were slightly reduce by 200 μM gentamicin in HeLa-Parental cells (**Figure 5B**). However, gentamicin significantly reduced the ATP-induced Ca^2+^ signals in HeLa-rCx26-GFP cells (**Figure 5D**). Therefore, the most frequent effect of gentamicin was a reduction in oscillation frequency (e.g., in HeLa-rCx26-GFP), thus, from the first Ca^2+^ rise to the return to baseline, cells oscillated an average of 8.8 ± 0.4 times in control conditions and were reduced to 5.1 ± 0.4 by 200 μM gentamicin (100 cells randomly analyzed in seven independent experiments). The AUC and duration of the ATP-elicited Ca^2+^ signals were significantly reduced by gentamicin in HeLa rCx26-GFP, but the reductions observed in HeLa-Parental cells were not statistically significant (**Figures 5E,F**). We also measured the Ca^2+^ signal after 20 min incubation with gentamicin, and no significant differences with respect to the values recorded after 5 min treatment were found (data not shown). Similar results were obtained in HeLa rCx26-GFP after 10 min preincubation with 100 μM CBX, a HC/GJC blocker, or 20 min preincubation with 200 μM GAP-26 (**Figures 5E,F**), a mimetic peptide that blocks Cx26 HCs (Evans and Leybaert, 2007). However, these blockers did not have significant effect on parental cells (**Figures 5E,F**).

It is known that extracellular ATP activates P2Y receptors coupled to G proteins, which in turn can activate PLC-dependent pathways, generating IP_3_ and thus promoting Ca^2+^ mobilization from the sarcoplasmic reticulum (Abbracchio et al., 2006). To determine whether this intracellular pathway is involved in the ATP-induced Ca^2+^ signal oscillations in HeLa-rCx26, we used a pharmacological approach. The possible involvement of PLC was tested using U73122 as inhibitor since, as mentioned above; PLC is a central component of the signal transduction mediated by activation of metabotropic purinergic receptors. To evaluate the participation of the endoplasmic reticulum in the ATP-induced Ca^2+^ signal oscillations, we used CPA, a sarcoplasmic-endoplasmic reticulum Ca^2+^-ATPase pump inhibitor (Suzuki et al., 1992). We determined the possible involvement of mitochondria in the ATP-induced Ca^2+^ signal oscillations (Ishii et al., 2006) in HeLa-rCx26 using the protonophore CCCP, which inhibits the mitochondrial Ca^2+^ transport by collapsing the proton electrochemical gradient (Bygrave, 1978).

**FIGURE 6:**
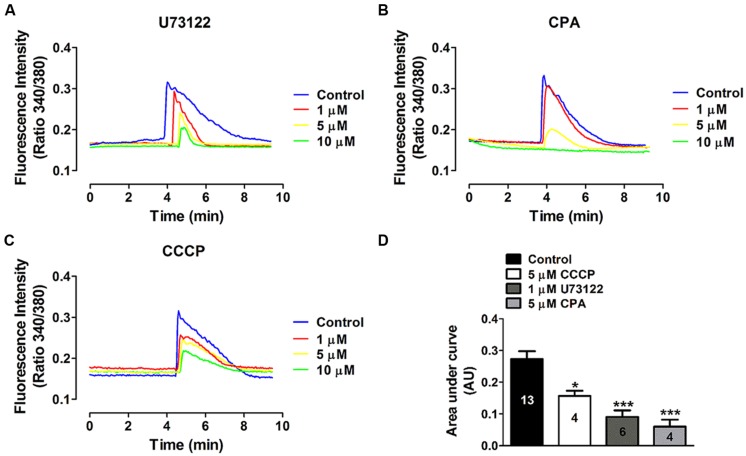
**Concentration-dependent effects of phospholipase C and intracellular Ca^2+^ stores inhibitors on the ATP-induced Ca^2+^ signal.** The effect of different concentrations of **(A)** U73122, a phospholipase C (PLC) inhibitor, **(B)** CPA, an endoplasmic reticulum Ca^2+^-ATPase pump inhibitor and **(C)** CCCP, a protonophore, was tested on the Ca^2+^ signal induced by 10 μM ATP. Representative average traces (including 40 cells per trace) measured in Fura-2 loaded HeLa-rCx26-GFP cells are shown. Cells were preincubated with each blocker during 10 min prior to stimulation with ATP. **(D)** Compiled data showing the amplitude of the ATP-evoked Ca^2+^ signals (mean ± SEM) measured as area under the curve and expressed as AU (mean ± SEM). Each trace represents the average from 40 cells per experiment. The number of independent experiments is indicated within each bar. *P < 0.05, ***P < 0.001.

**FIGURE 7:**
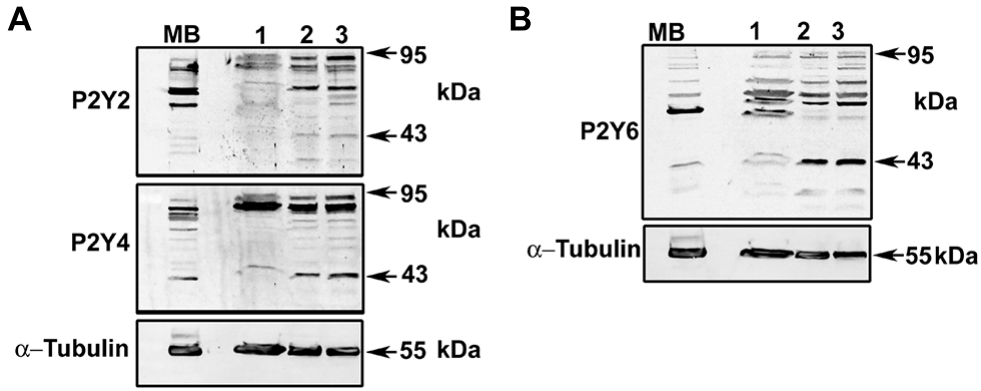
**Detection of P2Y_2_, P2Y_4_, and P2Y_6_ receptors in HeLa-Parental and HeLa-Cx26 cells.** Total homogenates of HeLa-Parental and HeLa cells expressing rCx26 or rCx26-GFP were resolved in sodium dodecyl sulfate polyacrylamide gel electrophoresis (12% SDS-PAGE). Membranes were probed with **(A)** anti-P2Y_2_R and anti-P2Y_4_R polyclonal or **(B)** anti-P2Y_6_R antibodies. Protein loading was controlled with anti-α-tubulin monoclonal antibody. Bands were detected with secondary anti-rabbit or anti-mouse antiserum coupled to horseradish peroxidase by electrogenerated chemiluminescence (ECL). MB, mouse brain (tissue lysate); Lane 1, HeLa-Parental; Lane 2, HeLa-rCx26; Lane 3, HeLa-rCx26-GFP.

To identify an optimal concentration at which the inhibitory effect was reproducible, the effect of different concentrations of each blocker was tested in Fura-2 loaded HeLa rCx26-GFP cells. **Figure 6** shows concentration-response experiments in which each blocker exhibited a concentration-dependent inhibition of ATP-induced Ca^2+^ signals. Neither U73122 nor CCCP eliminated completely the Ca^2+^ signals, but they strongly reduced them (**Figures 6A,C,D**). However, 10 μM CPA inhibited completely the ATP-induced Ca^2+^ signals (**Figures 6B,D**). These data indicate that extracellular ATP is acting through metabotropic receptors, activating PLC and releasing Ca^2+^ from the reticulum. To confirm these results, we tested whether HeLa cells express metabotropic purinergic receptors. Western blot analyses for P2Y receptors contained in whole HeLa-Parental and HeLa-Cx26 cell lysates were performed. We used polyclonal antibodies against different types of P2Y receptors (P2Y_2_R, P2Y_4_R, and P2Y_6_R) previously reported in HeLa cells (Okuda et al., 2003). Immunoreactive bands of ~34 and 95 kDa were detected for the three receptors in Hela-Parental cells and HeLa cells expressing rCx26 and rCx26-GFP, respectively (**Figure 7**). These bands were also detected in mouse brain (MB) lysate used as positive control. P2YRs receptors have been described to be 308–379 amino acid proteins with mass ranging from 41 to 53 kDa after glycosylation (D’Ambrosi et al., 2006). Bands with fast electrophoretic mobility were near the predicted molecular mass of P2Y_2_, P2Y_4_, and P2Y_6_ receptor protein subunits deduced from their cDNA sequence, being 42, 41 and 36 kDa, respectively (P41231, P51582, Q15077; SwissProtKB). These bands correspond to the monomeric forms while higher-order bands between 72 and 95 kDa may correspond to post-translational modified forms due to glycosylation (Sage and Marcus, 2002; Delbro et al., 2005), oligomeric forms of each receptor (D’Ambrosi et al., 2006, 2007) or heterodimerization between purinergic receptor subtypes (Yoshioka et al., 2001; Nakata et al., 2005; Suzuki et al., 2006). Notably, HeLa cells transfected with rCx26 or rCx26-GFP contained higher levels of the monomeric bands of each receptor protein than HeLa-Parental cells did (**Figures 7A,B**, 43 kDa). The functional expression of each receptor identified was also confirmed using a pharmacological approach with different purinergic agonists such as ATP, UTP, and UDP (data not shown).

### GENTAMICIN REDUCES ATP RELEASE TRIGGERED BY ACTIVATION OF P2Y RECEPTORS IN HeLa-rCx26 CELLS

**FIGURE 8:**
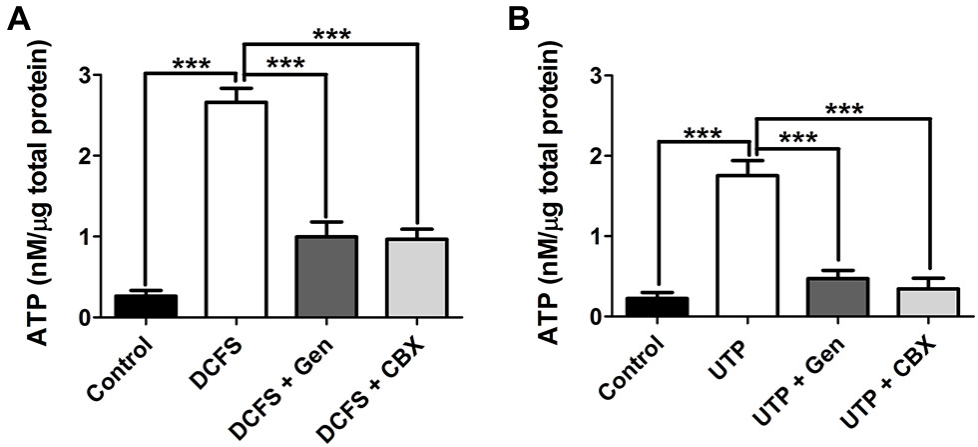
**Gentamicin reduces the ATP release induced by DCFS or UTP in HeLa-rCx26 cells.** ATP release from HeLa-rCx26 cells in response DCFS and purinergic receptor stimulation by UTP. ATP released to the extracellular solution was determined with an ATP bioluminescence assay kit 5 min after stimulation. **(A)** ATP released by HeLa-rCx26 cells exposed to DCFS, DCFS + 200 μM gentamicin (DCFS + Gen), DCFS + 200 μM CBX (DCFS + CBX) or with Ca^2+^/Mg^2+^ containing solution (control). **(B)** ATP released by HeLa-rCx26 stimulated with 100 μM UTP, 100 μM UTP + 200 μM gentamicin (UTP + Gen), 100 μM UTP +100 μM CBX (UTP + CBX) in Ca^2+^/Mg^2+^ solution (control). The data are shown as means [ATP] ± SEM of three independent experiments. ***P < 0.001.

Previous reports have shown that the expression of Cxs allows HeLa cells to release ATP in response to DCFS or purinergic receptor activation by UTP (Cotrina et al., 1998; De Vuyst et al., 2007). We studied the changes in extracelullar ATP using the luciferin-luciferase assay in response to DCFS to increase the open probability of rCx26 HCs in HeLa cells. In DCFS the amount of extracellular ATP was about 10-fold greater with respect to control conditions (Ca^2+^/Mg^2+^, **Figure 8A**). Additionally, the application of 200 μM gentamicin or 100 μM CBX reduced the DCFS-induced ATP release to values similar to those recorded in the presence of physiologic extracellular Ca^2+^/Mg^2+^ concentrations (**Figure 8A**). We also measured the release of ATP mediated by activation of P2Y purinergic receptors by extracellular 100 μM UTP in HeLa rCx26 in the presence of extracelullar 1.8 mM Ca^2+^ and 1 mM Mg^2+^. The ATP concentration increased about seven fold compared to basal release (without UTP, **Figure 8B**). Additionally the UTP-induced ATP release was drastically reduced by 200 μM gentamicin or 100 μM CBX (**Figure 8B**). These results suggest that gentamicin influences the amplitude, duration and shape of the ATP-induced Ca^2+^ signal, probably by decreasing the release of ATP through Cx HCs.

### GENTAMICIN DOES NOT INHIBIT INTERCELLULAR GAP JUNCTIONAL COMMUNICATION IN HeLa-rCx26 CELLS

**FIGURE 9:**
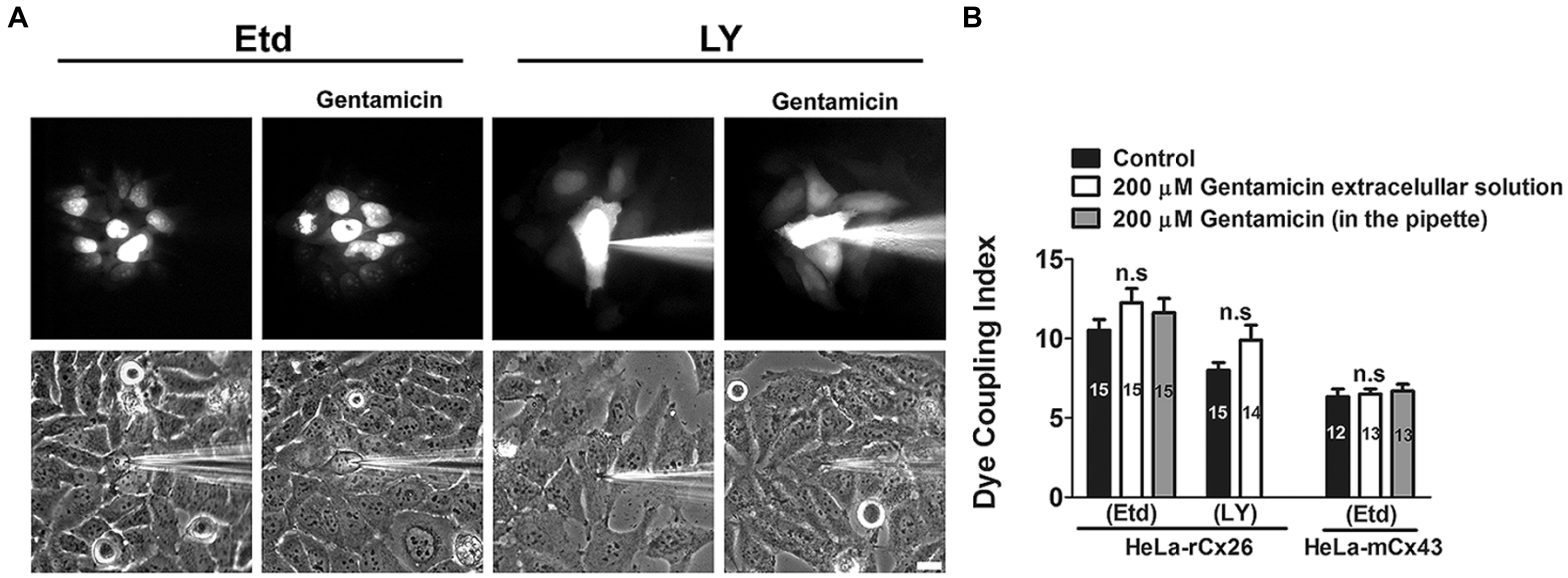
**Gentamicin does not inhibit the intercellular transfer of ethidium or Lucifer yellow between HeLa-rCx26 –mCx43 cells.** Gap junctional communication, expressed as dye coupling index, was evaluated in confluent cultures of HeLa-rCx26 or –mCx43 cells. A single cell was microinjected with ethidium (75 mM Etd in water) or Lucifer yellow (5% LY w/v in 150 mM LiCl) and the number of cells to which Etd or LY was transferred in 2 min was scored. Gentamicin was applied extracellularly or was included in the pipette at a final concentration of 200 μM. **(A)** Photomicrographs showing a fluorescent field of HeLa-xCx26 cells microinjected with Etd or LY and transfer to adjacent cells under control conditions and in the presence of 200 μM gentamicin applied extracellularly 5 min before the assay. Scale bar, 20 μm. **(B)** Quantitative analysis of dye coupling between HeLa-rCx26 and HeLa-mCx43 cells under control conditions (control) or treated with 200 μM gentamicin in the extracellular solution or in the pipette, as indicated. Bar graphs summarize the results of three independent experiments for each condition (the number of microinjections is indicated within each bar). Values correspond to the mean ± SEM of fluorescent cells.

We studied whether gentamicin inhibits GJCs in HeLa-rCx26 cells. To this end, dye coupling experiments were performed in confluent cultures. Single cells were microinjected with a solution containing Etd or LY, and the dye coupling index was scored in absence and presence of 200 μM gentamicin in the extracellular solution (**Figures 9A** and **8B**). Under control conditions, a mean of 10 ± 1 cells was scored. After 35 min exposure to 200 μM gentamicin, the dye coupling index tended to increase (12 ± 1 Etd coupled cells), although this response was not statistically significant compared to that of the control value (14 injected cells per experiment, in three independent cultures). Similar results were obtained using LY (from 8 ± 1 cells increased to 9 ± 1 cells after treatment with gentamicin; **Figures 9A,B**). In HeLa cells transfected with mCx43 the Etd coupling was not significantly affected by gentamicin applied in the extracellular solution (6 ± 1 cells under control conditions and 7 ± 1 cells after treatment with gentamicin; **Figure 9B**). When gentamicin was included in the pipette to a final concentration of 200 uM with the Etd solution, no significant differences were observed in the number of coupled cells in cultures of rCx26 or mCx43 (**Figure 9B**), indicating that gentamicin not affect the dye coupling when applied intracellularly.

## DISCUSSION

In this work, we found that the aminoglycoside antibiotic gentamicin applied extracellularly is a reversible Cx26 HC blocker that does not affect the functional state of gap junctions. Moreover, gentamicin drastically reduced both, the intracellular Ca^2+^ increase and the release of ATP induced by P2Y receptor activation by DCFS or UTP. Thus, the cellular toxicity of gentamicin might be explained partially by a perturbation of the autocrine/paracrine HC-dependent cell signaling, as in that mediated by extracellular ATP.

The addition of GFP to the C-terminus of rCx26 did not interfere with the HC blocking effect of gentamicin, suggesting that the C-terminal domain is not involved in gentamicin-induced inhibition of HCs. In addition, gentamicin also inhibited HCs formed by mCx43 or mCx45, which have longer C-terminus, indicating that its action is independent of the HC composition and the length of the C-terminus, a characteristic reported previously for other HC/GJC blockers (D’hondt et al., 2009; Wright et al., 2009). Our results differ from a previous study where rCx26 was shown not to form voltage-gated HCs in *Xenopus* oocytes and Neuro2A cells (González et al., 2006). The apparent discrepancy may be explained by differences in experimental procedures. González et al. (2006) used extracellular solution containing 1.8 mM Ca^2+^ and 0.8 mM Mg^2+^. In contrast, we used DCFS, which increases the open probability of all Cx HCs so far studied (Sáez et al., 2003). In agreement with our findings, activation of rCx26 HCs expressed in HeLa cells has been previously induced by DCFS (Jara et al., 2012).

Aminoglycosides block several ionic channels including hair cell METs (Jaramillo and Hudspeth, 1993), ACh receptors (Blanchet et al., 2000; Amici et al., 2005) and purinergic ionotropic channels (Bongartz et al., 2010). In most of these channels, gentamicin acts as open channel blocker (Kroese et al., 1989; Bongartz et al., 2010). Here, gentamicin was shown to inhibit Cx26 HC open either by DCFS or depolarization of the cellular membrane. Both conditions are known to increase the open probability of Cx26 HCs (Sánchez et al., 2010; Jara et al., 2012), and under these conditions we observed the most potent inhibitory effect of gentamicin. Thus, our data suggest that gentamicin could also act as open HC blocker.

In organotypic mouse cochlea, long distance propagation of intercellular calcium waves occurred through ATP released via Cx30 and Cx26 HCs, whereas GJCs composed by the same Cxs allow the simultaneous diffusion of IP_3_ across coupled cells (Anselmi et al., 2008). Moreover, ATP released through HCs located in cochlear supporting cells modulates the electromotility of the outer hair cells by activation of its ionotropic purinergic receptors that determine the cochlear sensitivity to sound stimulation in mammals (Zhao et al., 2005). Thus, altered purinergic signaling due to the blockade of HCs could have important consequences in auditory processing since purinergic transduction controls important aspects of the inner ear physiology, including the cochlear fluids homeostasis, essential for hearing (Housley and Gale, 2010). Relevant to this paracrine signaling, we found that gentamicin reduces the ATP-induced Ca^2+^ signal in HeLa-rCx26, which is in agreement with a previous report in which the ATP-induced Ca^2+^ transients of both HeLa-Cx26 and -Cx43 cells were inhibited by GAP26 and 18-βGA, two GJC/HC blockers. However, these compounds did not significantly affect the ATP-induced Ca^2+^ signal in HeLa-Parental cells (Verma et al., 2009). Our results demonstrate that activation of purinergic receptors by UTP increases the release of ATP in HeLa-rCx26 cells, which was inhibited by gentamicin and CBX. This is consistent with previous reports showing that the expression of Cxs allows HeLa cells to release ATP through HCs, as well as to mediate intercellular transfer of second messengers, such as IP_3_, and thereby generate larger Ca^2+^ waves (Cotrina et al., 1998; Paemeleire et al., 2000; Beltramello et al., 2005). Therefore, gentamicin could alter the purinergic signaling by blocking Cx26 HCs and consequently would reduce the ATP release. However, the Cx26 HC permeability to Ca^2+^ (Sánchez et al., 2010; Fiori et al., 2012) might also be involved in the ATP-induced Ca^2+^ signal.

HeLa cells have been shown to express low levels of functional P2X_7_Rs, which can be upregulated by pro-inflammatory cytokines, such as IFNγ (Welter-Stahl et al., 2009). However, and in agreement with Verma et al. (2009), our results in control conditions indicate that metabotropic purinergic receptors are the main mediators of the ATP-evoked Ca^2+^ signal in HeLa rCx26-GFP because they are strongly reduced by PLC inhibition and completely abrogated by sarcoplasmic-endoplasmic reticulum Ca^2+^-ATPase inhibition. Accordingly, it has been shown that ATP-evoked Ca^2+^ signals in HeLa cells are mediated by metabotropic receptors and are not significantly different from those exhibited by HeLa cells pre-treated with oATP, a P2X_7_R antagonist (Okuda et al., 2003; Welter-Stahl et al., 2009). In spite of the fact that gentamicin could block P2X_2_Rs (Bongartz et al., 2010), the previously mentioned results rule out the possibility that gentamicin affects the ATP-evoked signals by blocking ionotropic purinergic receptors. In support of this interpretation, a previous study has shown that P2X_2_R is not expressed in HeLa cells (Welter-Stahl et al., 2009). On the other hand, intercellular Ca^2+^ signals elicited through photo stimulation of caged IP_3_ propagate normally in cochlear organotypic cultures that lack P2X_7_Rs but fail to propagate in cultures with defective expression of Cx26 or Cx30 (Anselmi et al., 2008). Recently, Hu et al. (2012) reported that the expression of Cx26 increases in the cochlear lateral wall of rats 3 h after gentamicin administration, which could compensate for the loss of HC activity. It remains unknown how the overexpression of this protein is related to acquired hearing loss. In this line, we found that expression of Cx26 or Cx26-GFP enhanced the levels of the monomeric receptor. Changes in P2Y_1_R and P2Y_4_R levels in response to deletion of Cx43 in astrocytes have been described, and these changes in P2YRs also change the mode of propagation of intercellular Ca^2+^ signals (Suadicani et al., 2003). Moreover, other authors have suggested that Cx43 could interact directly with some purinergic receptors, including the P2Y_1_R (Iacobas et al., 2007). In light of the findings described above, we hypothesize that changes in the distribution of monomeric forms of purinergic receptors in response to Cx26 expression observed in the experiments described herein are due to direct interaction between Cx26 or Cx26-GFP with endogenously expressed purinergic receptors. However, future experiments are needed to address this issue. Our findings are relevant in the purinergic signaling context, because there is a growing amount of evidence showing that ATP released through HCs can trigger intercellular signaling, directly activating metabotropic and ionotropic purinergic receptors of cells in close contact, regulating both physiological and pathological processes in most tissues (Baroja-Mazo et al., 2013).

Finally, we found that gentamicin does not inhibit dye coupling via rCx26 or mCx43 GJCs when applied extracellularly. These results differ from those published by Todt et al. (1999) that showed inhibition of electrical gap junctional coupling induced by gentamicin in Hensen cells. Additionally, the inhibitory effect is time-dependent and the major effect was observed after 20 min treatment with gentamicin (Todt et al., 1999). In our experiments, cells were preincubated with gentamicin for only 5 min, and dye coupling was assessed during the following 30 min but no significant changes were observed. Relevant to this finding, it has been reported that some subtypes of HeLa cells are resistant to oxidative stress and grow at high oxygen concentrations due to potent antioxidant mechanisms (Campian et al., 2004). This feature might explain why we did not observe gentamicin-induced reduction in dye coupling as that observed in Hensen cells through an indirect mechanism involving production of free radicals and suppression by catalase (Todt et al., 1999). Another possibility could be that gentamicin requires access to the intracellular medium to block GJCs, however, we also performed experiments using gentamicin intracellularly through the microinjection pipette and we do not see significant changes in the dye coupling. Generally, an increase in gap junctional communication is associated with coordination of physiological responses (Ren et al., 1994; Mears et al., 1995; Curti and Pereda, 2004; Orellana et al., 2010; Johansen et al., 2011; Mhaske et al., 2013). In the present study, we did not find a statistical difference in the dye coupling index in presence of gentamicin in HeLa cells expressing rCx26 or Cx43. Therefore, we conclude that gentamicin applied extra or intracellularly does not change the dye coupling in HeLa transfected cells.

Recent studies have demonstrated that 24 h exposure to gentamicin 418, an antibiotic structurally and functionally similar to gentamicin, increases Cx43 phosphorylation and gap junction coupling in tubular proximal epithelial cell lines but decreases cell viability (Yao et al., 2010). Moreover, gain in HC activity has been associated with an increased incidence in cell death (Contreras et al., 2002; Orellana et al., 2010). Therefore, the gentamicin-induced closure might be linked to a deficient autocrine/paracrine signaling as discussed above, rather than the direct consequence of HC closure. Although gentamicin inhibits HCs and not GJCs, its use as HC blocker should be taken cautiously because of its pleiotropic effect on other membrane channels and its effect on free radical generation as discussed above.

Altogether, the data presented in this work suggest a new pharmacological target that could explain the deleterious side effects of gentamicin in the inner ear.

## CONCLUSION

In the present article, we report that extracellular gentamicin inhibits the activity of Cx26 HCs expressed in HeLa cells and *Xenopus* oocytes in a concentration-dependent manner without affecting the intercellular coupling through Cx26 GJCs. This effect does not exclusively require the *C*-terminus of Cx26 because it is also observed in HCs with other Cxs composition presenting a much longer C-terminal than Cx26. Finally and as described for other HC blockers, gentamicin reduced the ATP-induced Ca^2+^ signals.

## AUTHOR CONTRIBUTIONS

Vania A. Figueroa, Mauricio A. Retamal, Agustín D. Martínez, and Juan C. Sáez designed research; Vania A. Figueroa, Mauricio A. Retamal, Luis A. Cea, José D. Salas, Aníbal A. Vargas, Christian A. Verdugo, and Oscar Jara performed research; Vania A. Figueroa, Mauricio A. Retamal, Luis A. Cea, José D. Salas, Aníbal A. Vargas, Christian A. Verdugo analyzed data; and Vania A. Figueroa, Mauricio A. Retamal, Agustín D. Martínez, and Juan C. Sáez wrote the paper.

## Conflict of Interest Statement

The authors declare that the research was conducted in the absence of any commercial or financial relationships that could be construed as a potential conflict of interest.

## ABBREVIATIONS

CCCP: carbonyl cyanide 3-chlorophenylhydrazone
CPA: cyclopiazonic acid
Cxs: connexins
Etd: ethidium
GJCs: gap junction channels
HCs: hemichannels.
